# Post-translational Modifications of the Peptidyl-Prolyl Isomerase Pin1

**DOI:** 10.3389/fcell.2020.00129

**Published:** 2020-03-04

**Authors:** Dongmei Chen, Long Wang, Tae Ho Lee

**Affiliations:** Fujian Key Laboratory for Translational Research in Cancer and Neurodegenerative Diseases, Institute for Translational Medicine, School of Basic Medical Sciences, Fujian Medical University, Fuzhou, China

**Keywords:** Pin1, phosphorylation, oxidation, post-translational modification, SUMOylation, ubiquitination, Alzheimer’s disease, cancer

## Abstract

The peptidyl-prolyl *cis/trans* isomerase (PPIase) Pin1 is a unique enzyme that only binds to Ser/Thr-Pro peptide motifs after phosphorylation and regulates the conformational changes of the bond. The Pin1-catalyzed isomerization upon phosphorylation can have profound effects on substrate biological functions, including their activity, stability, assembly, and subcellular localization, affecting its role in intracellular signaling, transcription, and cell cycle progression. The functions of Pin1 are regulated by post-translational modifications (PTMs) in many biological processes, which include phosphorylation, ubiquitination, SUMOylation and oxidation. Phosphorylation of different Pin1 sites regulates Pin1 enzymatic activity, binding ability, localization, and ubiquitination by different kinases under various cellular contexts. Moreover, SUMOylation and oxidation have been shown to downregulate Pin1 activity. Although Pin1 is tightly regulated under physiological conditions, deregulation of Pin1 PTMs contributes to the development of human diseases including cancer and Alzheimer’s disease (AD). Therefore, manipulating the PTMs of Pin1 may be a promising therapeutic option for treating various human diseases. In this review, we focus on the molecular mechanisms of Pin1 regulation by PTMs and the major impact of Pin1 PTMs on the progression of cancer and AD.

## Introduction

Post-translational modifications (PTMs) of proteins play important roles in regulating protein conformation, localization, stability, and activity and ultimately induce a number of fundamental biological functions, including signal transduction, protein–protein interaction, protein trafficking, cell differentiation, and proliferation (Marcelli et al., 2018; Wu et al., 2019). To date, more than 450 PTMs have been identified, including phosphorylation, oxidation, ubiquitination, and SUMOylation (Venne et al., 2014). PTMs are reversible and tightly regulated during physiological conditions. However, gene mutations, increased cellular stresses, and deregulated cellular signals can modify PTMs or introduce non-specific PTMs and contribute to the development of human disease, notably cancer and neurodegeneration (Martin et al., 2011; Mowen and David, 2014; Marcelli et al., 2018; Wu et al., 2019).

**FIGURE 1:**
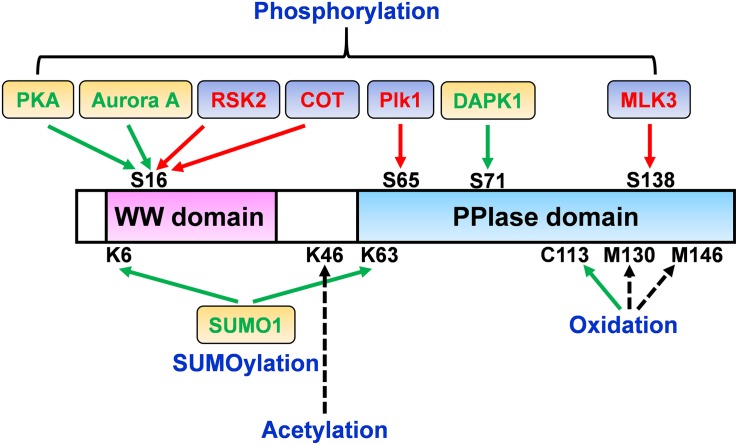
Pin1 regulation perspective by post-translational modifications (PTMs). The molecular mechanisms and activity/function of Pin1 can be regulated by multiple signals through a variety of PTMs. While the phosphorylation of Ser16 in the WW domain of Pin1 by RSK2 and COT increases Pin1 functions, Ser16 phosphorylation by PKA and Aurora A negatively regulates Pin1 activity. Ser65 phosphorylation in the PPIase domain by Plk1 prevents Pin1 ubiquitination and increases its stability. Moreover, DAPK1 directly binds and phosphorylates Pin1 on Ser71, which suppresses Pin1 activity and functions. Furthermore, Pin1 phosphorylation at Ser138 by MLK3 increases Pin1 nuclear localization and functions. Pin1 SUMOylation in both the WW and PPIase domains (K6 and K63) decreases Pin1-induced proliferation and cell transformation. Pin1 oxidation on C113 is highly increased in the human AD brain and abolishes Pin1 enzymatic activity. Pin1 acetylation on Lys46 has been identified in human acute myeloid leukemia and neuroblastoma cell lines. The regulatory role of Pin1 acetylation on Lys46 and oxidation on Met130 and Met146 remains to be determined. Green arrow, positive regulation of Pin1 activity; red arrow, negative regulation of Pin1 activity; black dotted arrow, unknown.

The peptidyl-prolyl *cis/trans* isomerase (PPIase) Pin1 was first identified by a combined genetic and biochemical screening strategy based on its physical interaction with the *Aspergillus* mitotic kinase NIMA, and the function of which Pin1 suppresses to induce mitotic catastrophe (Lu et al., 1996; Lu and Zhou, 2007; Zhou and Lu, 2016). Pin1 is a unique prolyl isomerase that specifically binds and isomerizes certain phosphorylated serine or threonine residues preceding proline (pSer/Thr-Pro) (Yaffe et al., 1997; Lu et al., 1999b; Lu and Zhou, 2007; Zhou and Lu, 2016). Pin1 induces conformational changes in phosphorylated target proteins because pSer/Thr-Pro motifs exist in two distinct *cis* and *trans* conformations (Yaffe et al., 1997; Lu et al., 2007). Pin1-induced conformational changes have been shown to play a crucial role in the cellular functions, including the cell cycle, cell signaling, transcription and splicing, DNA damage responses, germ cell development, and neuronal survival (Crenshaw et al., 1998; Shen et al., 1998; Fujimori et al., 1999; Winkler et al., 2000; Zhou et al., 2000; Wulf et al., 2001, 2002; Liou et al., 2002; Zacchi et al., 2002; Zheng et al., 2002; Atchison and Means, 2003; Atchison et al., 2003; Xu et al., 2003; Lu and Zhou, 2007; Moretto-Zita et al., 2010; Lee et al., 2011b). The expression and function of Pin1 are tightly controlled at multiple levels by transcriptional, post-transcriptional, and post-translational regulation under physiological conditions. In particular, Pin1 deregulation including PTMs is directly involved in an increasing number of pathological conditions, notably premature aging, cancer and Alzheimer’s disease (AD) (Lu et al., 1999a; Ryo et al., 2001, 2002; Liou et al., 2003; Bao et al., 2004; Lu, 2004; Akiyama et al., 2005; Pastorino et al., 2006, 2012, 2013; Suizu et al., 2006; Yeh et al., 2006; Balastik et al., 2007; Lu and Zhou, 2007; Takahashi et al., 2007, 2008; Yeh and Means, 2007; Lee et al., 2009, 2011b; Teng et al., 2011; Nakatsu et al., 2016; Zhou and Lu, 2016; Han et al., 2017). This review focuses on the molecular mechanisms of Pin1 regulation by PTMs and discusses the major impact of Pin1 deregulation on the progression of cancer and AD (Figure 1).

## Pin1 Structure

The human Pin1 is composed of 163 amino acids with a mass of 18 kDa (Lu et al., 1996). Pin1 contains two distinct major domains: an N-terminal WW domain (residues 1–39) and a C-terminal PPIase domain (residues 50–163), which are connected by a flexible linker region (residues 35–53) (Ranganathan et al., 1997; Lu et al., 1999b; Lu and Zhou, 2007; Lee and Liou, 2018). In addition, an interdomain interface between the two domains, consists of WW domain Loop II (residues 27–30) and part of the PPIase domain (residues 138–142). This domain interface has been reported to play an important role in the allosteric regulation of Pin1 functions (Namanja et al., 2007, 2011; Wilson et al., 2013).

The WW domain consists of a triple-stranded anti-parallel β-sheet and two conserved tryptophan residues that are essential for binding to the phosphorylated proteins (Ranganathan et al., 1997; Lee and Liou, 2018). Thus, the WW domain acts on the pSer/Thr-Pro motif binding module, which targets the Pin1 catalytic domain close to the substrate binding sites, where the PPIase domain isomerizes specific pSer/Thr-Pro motifs to induce conformational changes, in a type of “double-check” mechanism (Lu et al., 1996, 1999b, 2007; Ranganathan et al., 1997; Zhou et al., 2000; Lu and Zhou, 2007; Lee et al., 2011b). Although it is believed that the combined primary structure with sequence-specific dynamics is important for WW domain substrate specificity, it remains unclear why Pin1 binds only to specific pSer/Thr-Pro motifs in certain proteins.

The PPIase domain contains a PPIase binding domain that can bind to pSer/Thr-Pro motifs and a catalytic loop at the catalytic site (Ranganathan et al., 1997; Lee and Liou, 2018). A hydrophobic pocket within the PPIase domain is composed of the substrate proline that binds through the cyclic side chain of residues Leu122, Met130, and Phe134, and the peptidyl-prolyl bond that catalyzes the *cis*/*trans* isomerization surrounded by side chain residues His59, Cys113, Ser154, and His157 (Ranganathan et al., 1997). A phosphate-binding loop that undergoes substrate recognition consists of the residues Lys63, Arg68, and Arg69, and mediates the catalytic selectivity for the N-terminal side chain binding to the proline (Ranganathan et al., 1997; Behrsin et al., 2007).

Moreover, the WW domain can regulate PPIase activity depending on whether a peptide substrate is phosphorylated on a single site or on multiple sites. Subsequent studies have shown that most Pin1 substrates contain a single phosphorylation target for the WW domain and that the PPIase domain would have to act on the same pSer/Thr-Pro motif to accelerate its isomerization (Yaffe et al., 1997; Lu et al., 1999b; Lu and Zhou, 2007; Peng et al., 2007). For example, a Thr668-Pro motif in an amyloid precursor protein (APP) exists in the *trans* conformations before phosphorylation, as indicated through nuclear magnetic resonance (NMR) analysis (Pastorino et al., 2006). However, the *cis* conformation appears only after phosphorylation due to the limitations imposed by the local structure (Ramelot and Nicholson, 2001). Pin1 binds to the pThr668-Pro motif in APP, which accelerates APP isomerization to the *trans* configuration, which results in the suppression of amyloidogenic APP processing and amyloid-β production (Pastorino et al., 2006, 2012; Ma et al., 2012) although there are conflicting results (Akiyama et al., 2005). Alternatively, Pin1 binds to multiple pSer/Thr-Pro motifs in a single substrate. Pin1 binds to phosphorylated p53 on Ser33 and Ser46 in response to DNA damage, and regulates the stability of p53 (Wulf et al., 2002; Zacchi et al., 2002; Zheng et al., 2002). Consistently, it has been found that both Ser33 and Ser46 residues are close to the MDM2-binding site and affect transcriptional activity of p53 (Kussie et al., 1996; Haupt et al., 1997; Kubbutat et al., 1997), suggesting that Pin1 binds to and isomerizes p53 on both the phosphorylated Ser33 and Ser46 sites. The isomerization may suppress the interaction of p53 with its ubiquitin ligase MDM2, affect the phosphorylation of p53 at other sites, and/or affect the p53 transcriptional mediation of p21. The evidence that Pin1 substrates may have multiple phosphorylation sites or form a multi-protein complex suggests that the WW domain and PPIase domain might act on different pSer/Thr-Pro motifs in the same protein or in different proteins. Further studies are needed to solve these important questions.

## Dual Roles of Pin1 in the Development of Cancer and Ad

Although cancer represents proliferating characteristics and AD shows degenerating features, two distinct diseases share common signaling mechanisms, including Pro-directed phosphorylation regulation (Driver and Lu, 2010). Pin1 has been shown to promote cell proliferation and has a protective role against neurodegeneration including AD, however, Pin1 exerts opposite effects on the development of cancer and AD (Yeh and Means, 2007; Takahashi et al., 2008; Zhou and Lu, 2016; Han et al., 2017).

In cancer, Pin1 expression and activity are aberrantly elevated in many malignancies, which are regulated in genetic, transcriptional, post-transcriptional, and PTMs levels (Bao et al., 2004; Takahashi et al., 2008; Lee et al., 2011b, 2014; Li et al., 2013; Lu and Hunter, 2014; Luo et al., 2014; Zhou and Lu, 2016). Upregulated Pin1 controls many Pro-directed phosphorylation signaling events and its modulation is involved in cell cycle coordination, chromosome instability, proliferation, migration, metastasis, and apoptosis in cancer cells (Zhou and Lu, 2016). Indeed, Pin1 is known to activate more than 50 oncogenes or growth-promoting regulators and suppress a number of suppressors or growth inhibitory regulators by regulating activity, protein interaction, stability, and cellular localization (Min et al., 2016). Moreover, Pin1 has been shown to increase self-renewal activity and promote breast cancer stem cell-mediated tumorigenesis (Luo et al., 2014, 2015; Rustighi et al., 2014). In animal models, Pin1 deficiency effectively prevents tumorigenesis by overexpressing Neu, but not c-Myc, and Pin1 overexpression in mammary gland induces chromosome instability and leads to malignant breast cancer (Wulf et al., 2004; Suizu et al., 2006). Furthermore, double Pin1 and p53 knockout (KO) mice are completely resistant to tumorigenesis although these mice show increased levels of thymic hyperplasia (Takahashi et al., 2007).

Although cumulative results suggest that Pin1 is strongly associated with cell proliferation and cancer development, Pin1 has been shown to have a tumor suppressor function (Yeh and Means, 2007; Takahashi et al., 2008; Han et al., 2017). It has been reported that the expression levels of Pin1 are downregulated in renal cell carcinoma due to gene deletion and Pin1 restoration reduces tumor growth of human renal cell carcinoma cells (Teng et al., 2011). Moreover, Pin1 ablation in mouse embryonic fibroblasts of C57BL6 background has been shown to increase cyclin E stability and accelerate genomic instability (Yeh et al., 2006). Furthermore, Pin1 stabilizes tumor suppressor p53 (Wulf et al., 2002; Zacchi et al., 2002; Zheng et al., 2002) and inactivate oncoprotein c-Myc (Yeh et al., 2004) although the same group has reported that Pin1 acts as a transcriptional coactivator for c-Myc and increases its tumorigenic activity (Farrell et al., 2013). These results suggest that Pin1 might be a conditional tumor suppressor in a certain context depending on genetic background, tissue and upstream regulation of Pin1, such as PTMs. More studies are needed to clarify the molecular mechanisms by which Pin1 has opposite effects on the development of cancer.

One of major features of AD is aggregated neurofibrillary tangles that consist of hyperphosphorylated tau (Binder et al., 2005; Ballatore et al., 2007). The phosphorylated forms of tau are dissociated from microtubules and disrupt microtubule structure integrity (Geschwind, 2003). Pin1 specifically binds to phosphorylated tau on Thr231-Pro motif and promotes its *cis* to *trans* conformation (Lu et al., 1999a; Liou et al., 2003). *Cis* phosphorylated tau, but not *trans*, appears very early in human brains with mild cognitive impairment (MCI), tends to be aggregated, and is associated with neurofibrillary degeneration (Nakamura et al., 2012a). Therefore, Pin1-catalyzed the isomerization of phosphorylated tau restores its ability to promote microtubule assembly and may prevent Alzheimer’s tau pathology. Among sporadic AD patients, accumulation of senile plaques composed of amyloid beta (Aβ) peptides which derives from APP is regarded as another pathological hallmark (Tanzi and Bertram, 2005; Thinakaran and Koo, 2008). APP is known to be processed in two sequential cleavages by β-secretase in the extracellular domain of the full-length APP and γ-secretase in the transmembrane region, releasing the intact Aβ during the development of AD (Thinakaran and Koo, 2008). Cdk5- and GSK3β-mediated Thr668 phosphorylation may facilitate APP cleavage by β-secretase, thereby increasing Aβ secretion (Lee et al., 2003; Phiel et al., 2003; Cruz et al., 2006). Importantly, Pin1 binds to the phosphorylated Thr668-Pro motif of full length APP and accelerates the isomerization from *cis* to *trans* by over 1000-fold (Pastorino et al., 2006). Overexpression of Pin1 decreases Aβ secretion *in vitro* whereas Pin1 ablation in mice promotes amyloidogenic APP processing and increases insoluble toxic Aβ42 in an age-dependent manner (Pastorino et al., 2006, 2012; Ma et al., 2012). However, Akiyama et al. (2005) found that Pin1 binds to phosphorylated Thr668 of β-secretase-cleaved C-terminus APP product, C99 rather than full length APP and promotes Aβ production in the mouse embryonic fibroblasts or COS7 cells. Moreover, soluble and insoluble Aβ levels are decreased in Pin1 deficient mice compared with WT mice (Akiyama et al., 2005). Since Pin1 is prominently localized with full length APP at the plasma membrane and presumably binds to C99 in the cytosol, these opposite results might be due to Pin1 cellular compartment, different APP metabolism, and/or feedback mechanisms (Takahashi et al., 2008; Pastorino et al., 2013). Further studies are needed to elucidate molecular mechanisms by which Pin1 regulates Aβ production and its binding capacity of different APP cleavage products in the development of AD.

## Post-Translation Modifications of Pin

### Phosphorylation of Pin1

Protein phosphorylation on Ser/Thr-Pro is a critical signaling mechanism in regulating many cellular processes by causing changes in protein conformation and its deregulation contributes to many human diseases including cancer and AD (Blume-Jensen and Hunter, 2001; Pawson and Scott, 2005; Olsen et al., 2006; Lee et al., 2011b). Pin1, including the WW and PPIase domains, is phosphorylated at multiple sites, and this phosphorylation regulates its binding ability, enzymatic activity, and function in both physiological and pathological conditions (Lu and Zhou, 2007).

**FIGURE 2:**
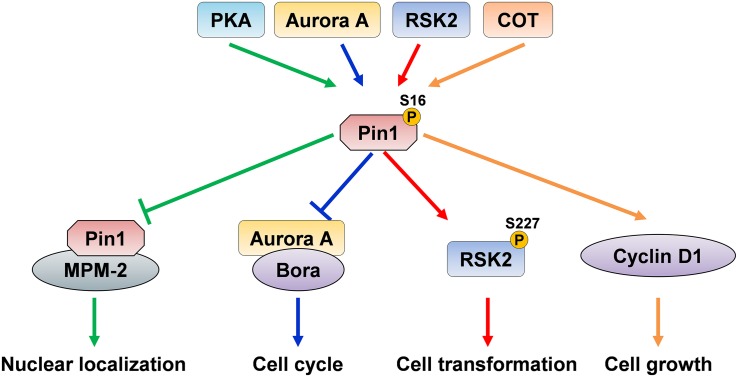
The dual effects of Pin1 Ser16 phosphorylation. Pin1 phosphorylation on Ser16 by PKA inhibits the binding ability of Pin1 to MPM-2, thereby suppressing Pin1 nuclear localization. Aurora A directly binds to and phosphorylates Pin1 and suppresses its binding to Bora, and ultimately disrupts cell cycle, indicating that PKA and Aurora A inhibit Pin1 ability to interact with its substrate and function. However, Pin1 phosphorylation by RSK2 induces RSK2 phosphorylation and promotes cell transformation. COT directly phosphorylates Ser16 of Pin1 and increases cyclin D1 levels, and subsequently promotes cell growth, suggesting that RSK2- and COT-mediated Pin1 phosphorylation increase Pin1-induced cell proliferation.

It has been reported that cAMP-protein kinase A (PKA) phosphorylates Pin1 at Ser16 in the WW domain *in vitro* and *in vivo* (Lu et al., 2002). The phosphorylation of Ser16 abolishes the ability of Pin1 to interact with its substrate, MPM-2 antigen, and disrupts Pin1 nuclear speckle localization. Since Ser16 is one of the important amino acid residues which are responsible for the binding of phosphorylated substrate to the WW domain, this modification may affect the binding ability of Pin1 and its functions (Lu et al., 2002). Aurora A can directly interact with and phosphorylate Pin1 at Ser16 during G2/M progression, which markedly suppresses the function of Pin1 in G2/M, thus affecting the cell cycle (Lee et al., 2013). Pin1 overexpression also delays mitotic entry by inducing the premature degradation of Aurora A cofactor Bora in the G2 phase through the β-TrCP-mediated ubiquitin-proteasome pathway and alters the cytoplasmic translocation of endogenous Bora. However, Pin1 phosphorylation on Ser16 by Aurora A disrupts its binding ability to Bora, which increases Bora protein stability and ultimately suppresses mitotic progression (Lee et al., 2013). Since Pin1 plays a critical role as a cell cycle modulator to promote cell cycle progression and since cell cycle disorder is a common phenomenon in cancer (Lin et al., 2015), phosphorylation of Pin1 at Ser16 might be tightly regulated during the cell cycle such that its deregulation might cause cell cycle disruption in pathological conditions. While PKA- or Arora A-mediated Ser16 phosphorylation abolishes the ability of Pin1 to bind to its substrates, other kinases increase Pin1 function and/or binding ability through Ser16 phosphorylation. Ribosomal protein S6 kinase 2 (RSK2) forms a strong complex with Pin1 and phosphorylates Ser16 (Cho et al., 2012). Moreover, 12-O-tetradecanoylphorbol-13-acetate (TPA) promotes the interaction of RSK2 and Pin1, which promotes RSK2 phosphorylation, thereby increasing TPA-induced cell transformation (Cho et al., 2012). In addition, the MAP3K-related serine/threonine kinase COT has been reported to directly phosphorylate Pin1 at Ser16 in the presence of TPA (Kim et al., 2015). COT overexpression leads to Pin1 phosphorylation, which subsequently increases cyclin D1 abundance and enhances mammary gland tumorigenesis in MCF7 cells. Consistently, tumor growth has been abrogated in a nude mouse xenograft model treated with Pin1 inhibitor and/or COT kinase inhibitor. Furthermore, Pin1 pSer16 levels have been shown to be positively correlated with COT levels in human breast cancer (Kim et al., 2015). These opposite results of Pin1 Ser16 phosphorylation might be due to responsible kinases, different cellular locations, the selected cell lines or differences in the experimental sensitivity and specificity of the methods used (Figure 2). In addition, tau and GSK3β dephosphorylation promotes Pin1 phosphorylation at Ser16, suggesting that Ser16 phosphorylation of Pin1 in the brain might have a protective role against AD (Min et al., 2005). However, Ando et al. (2013) showed that the level of Ser16 phosphorylation of Pin1 is highly increased in human AD brain tissues compared to those of normal subjects. Therefore, the *in vivo* effects and mechanisms of Pin1 Ser16 phosphorylation on its substrate binding ability and function remain to be determined.

Phosphorylation in the PPIase domain also regulates Pin1 functions. Eckerdt et al. (2005) showed that Polo-like kinase 1 (Plk1), a critical regulator of mitosis, phosphorylates Pin1 at Ser65 in the PPIase domain. However, neither the phosphorylation of Pin1 by Plk1 nor mimicked phosphorylation at Ser65 has an effect on the catalytic activity of Pin1. Interestingly, phosphorylation of Pin1 by Plk1 enhances Pin1 stability by inhibiting its ubiquitination. In addition, inhibition of Plk1 activity by transfection of siRNA targets to Plk1 or by transfection of dominant negative Plk1 K82M or hyperactive Plk1 T210D enhances the ubiquitination of Pin1. Ubiquitination is the conjugation of proteins and ubiquitin, a highly conserved 76-amino-acid eukaryotic protein, and is essential for the degradation of proteins (Schlesinger and Goldstein, 1975; Ciechanover et al., 1980a, b; Hershko et al., 1980; Weissman, 2001; Pickart and Eddins, 2004). Aberrant ubiquitination impacts a wide range of eukaryotic biology, and its defective regulation results in extensive developmental diseases, including cancer and neurodegenerative diseases (Rape, 2018). Plk1 expression is dramatically upregulated in tumor cell lines and in human cancer tissues (Yuan et al., 1997; Liu et al., 2017). Since overexpression of Pin1 contributes to tumorigenesis, these observations indicate that the stabilization of Pin1 might be one of the mechanisms that contribute to Plk1-mediated tumorigenesis. However, it remains unclear whether Plk1 directly phosphorylates Pin1 *in vivo*.

**FIGURE 3:**
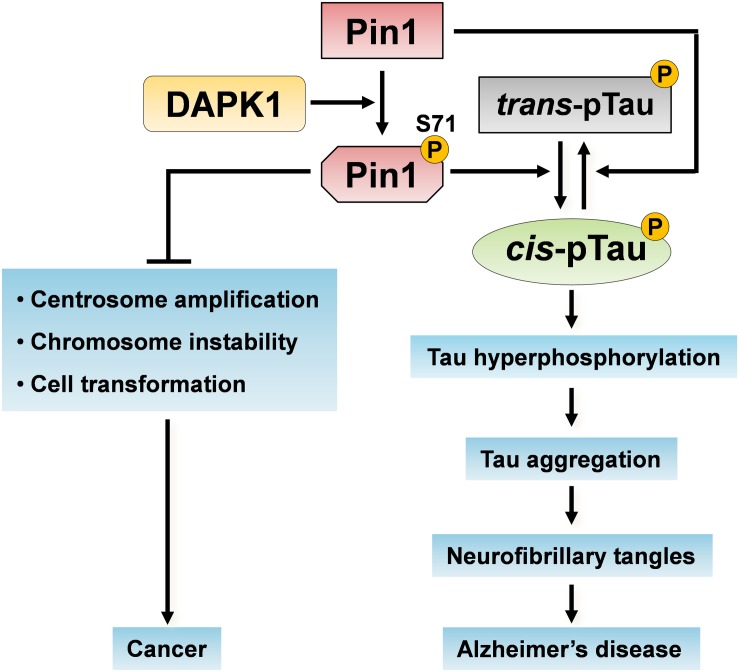
Pin1 phosphorylation on Ser71 by DAPK1 in cancer and AD. The activation of DAPK1 directly phosphorylates Pin1 on Ser71 and inhibits Pin1 functions. Pin1 phosphorylation on Ser71 inhibits its catalytic activity and the ability of Pin1 to induce centrosome amplification, chromosome instability and cell transformation, thereby suppressing oncogenic pathways. Moreover, the inactivation of Pin1 promotes the *trans* p-tau to *cis* p-tau conversion, induces tau hyperphosphorylation and aggregation, which might eventually cause neurofibrillary tangles in the development of AD.

Death-associated protein kinase 1 (DAPK1) directly binds to and phosphorylates Pin1 on Ser71, which is located in the substrate binding loop consisting of residues 63–80 in the PPIase domain (Bialik and Kimchi, 2011; Lee et al., 2011a, b). DAPK1 is a calcium/calmodulin-dependent Ser/Thr kinase that is involved in cell death, and its deregulation is implicated in cancer and AD (Cohen et al., 1997; Inbal et al., 1997; Kissil et al., 1997; Kim et al., 2014, 2016, 2019; Zhao et al., 2015; You et al., 2017; Chen et al., 2019). Pin1 phosphorylation on Ser71 by DAPK1 inhibits the catalytic activity of Pin1, specifically suppressing the ability of Pin1 to activate transcription factors and stabilize proteins, blocks Pin1 nuclear localization, and attenuates the centrosome amplification, chromosome instability and cell transformation induced by Pin1 (Lee et al., 2011a). Since Ser71 is located at the center of the PPIase domain binding pocket, this phosphorylation might prevent phosphorylated substrates to enter the catalytic active site (Lee et al., 2011a; Mahoney et al., 2018). Furthermore, DAPK1 increases tau protein stability and phosphorylation through Pin1 Ser71 phosphorylation (Kim et al., 2014). Pin1 ablation or inhibition induces the development of AD pathologies (Liou et al., 2003; Pastorino et al., 2006; Lu and Zhou, 2007; Lim et al., 2008), suggesting that aberrant DAPK1 activation might contribute to the age-dependent neurodegeneration in AD by inhibiting Pin1 function. Therefore, DAPK1 might be involved in the development of cancer and AD by regulating Pin1 function through its phosphorylation of Ser71 (Figure 3).

In contrast, Pin1 phosphorylation of Ser138 in the PPIase domain by mixed-lineage kinase 3 (MLK3) enhances the functions of Pin1 by increasing its catalytic activity and nuclear translocation (Rangasamy et al., 2012). The Ser138 phosphorylation of Pin1 promotes cell-cycle progression, cyclin D1 protein stability, and centrosome amplification. In breast cancer tissues, the levels of Pin1 phosphorylated at Ser138 are significantly upregulated, indicating that targeting MLK3 or Pin1 Ser138 might benefit cancer treatment (Rangasamy et al., 2012). Thus, Pin1 phosphorylation in both the WW and PPIase domains regulates its substrate binding ability, subcellular localization, and function, and this modification might contribute to tumorigenesis and neurodegeneration.

### Oxidation of Pin1

Oxidative stress has been widely regarded as a contributing factor to AD and cancer, as indicated by protein oxidation, lipid peroxidation, nucleic acid oxidation, advanced glycation products, and reactive oxygen species (ROS) formation (Butterfield and Lauderback, 2002; Zhu et al., 2004; Gorrini et al., 2013; Tonnies and Trushina, 2017). It has been reported that Pin1 oxidation levels are significantly increased in patients with MCI and that Pin1 catalytic activity is decreased in the hippocampus region of their brains (Butterfield et al., 2006; Sultana et al., 2006). These results suggest that Pin1 is likely involved in the initial development of MCI to AD because MCI is an intermediate stage between normal cognitive aging and early dementia or clinically probable AD, and which eventually develops AD. Indeed, Pin1 has been found to be oxidized in the brains of AD patients, and Pin1 oxidation appeared to decrease Pin1 activity by reducing its isomerase activity (Butterfield et al., 2006; Sultana et al., 2006). Moreover, oxidized Pin1 could be recognized by the ubiquitination system for its degradation (Tramutola et al., 2018). However, the site(s) where Pin1 is oxidized was unknown, and how this oxidative modification affects Pin1 catalytic activity was unclear. Recently, two independent studies have identified the oxidation site in Pin1 by mass spectrometry and X-ray crystallization (Aluise et al., 2013; Chen et al., 2015). Pin1 is modified by an oxidative modification of Cys113 in the PPIase domain upon hydrogen peroxide (Chen et al., 2015). Although Pin1 that is oxidized on Cys113 can still effectively bind to substrates, Pin1 enzymatic activity is abolished, indicating that it can trap substrates. Moreover, Pin1 oxidation has been found to inhibit Pin1 nuclear localization, and increase tau/APP protein stability and Aβ secretion, and it is increased in human AD brains, as well as in AD mouse models (Chen et al., 2015). Thus, Pin1 oxidation of Cys113 causes Pin1 inactivation and mislocalization, thereby contributing to the development of AD. Other oxidative modification sites in Pin1 have been identified at Met130 and Met146 in human neuroblastoma SH-SY5Y cells stably expressing Pin1, indicating that Pin1 might have multiple oxidation sites through which its function is regulated (Ando et al., 2013).

### SUMOylation of Pin1

A small ubiquitin-like modifier (SUMO) peptide on a lysine residue plays important roles in regulating a spectrum of protein functions, including protein activity, stability, and localization (Geiss-Friedlander and Melchior, 2007; Gareau and Lima, 2010; Hannoun et al., 2010). The SUMOylation and deSUMOylation of proteins is a highly dynamic process, and only a small fraction of a substrate is modified at a given time. Increasing evidence indicates that deregulation of either SUMO conjugation or deconjugation can contribute to tumorigenesis (Eifler and Vertegaal, 2015). Chen and coworkers found that Pin1 is SUMOylated on Lys6 in the WW domain and on Lys63 in the PPIase domain, as determined by mass spectrometric analysis and site-directed mutagenesis assay (Chen et al., 2013). Both Lys6 and Lys63 in Pin1 resemble the consensus SUMOylation site. Among mammalian SUMO isoforms such as SUMO1, SUMO2, and SUMO3, only SUMO1 specifically promotes SUMOylation of Pin1 at both sites. SUMOylation inhibits the substrate binding ability, phospho-specific PPIase activity, and cellular function of Pin1. Since SUMO1 that is conjugated at Lys6 is very close to the Trp34 residue of the WW domain, which is essential for Pin1 to bind to its phosphorylated substrates, and Lys63 is critical for anchoring the pSer/Thr-binding pocket in the PPIase domain, these modifications might affect Pin1 substrate binding and catalytic activity. Moreover, SUMO protease (SENP) 1 directly binds to the PPIase domain of Pin1 and promotes deSUMOylation of Pin1 after exposure to oxidative stress, suggesting that SENP1 may reverse Pin1 inhibition that had been induced by SUMOylation. Indeed, SENP1 promotes Pin1-induced centrosome amplification, chromosome instability, proliferation, and cell transformation (Chen et al., 2013). These results further demonstrate that SENP1 levels are positively correlated with Pin1 levels in human breast cancer tissues. Thus, in cancers, modifications of Pin1 by SENP1 may contribute to cell proliferation and tumorigenesis.

## Therapeutic Targeting of Pin1 Ptms

Since deregulation of Pin1 expression and activity has critical effects on the development of cancer and AD, and Pin1 has a specified substrate binding and active site, targeting of Pin1 has been an attractive druggable target. The widely used Pin1 inhibitors such as juglone, PiB, pTide, and TME-001, have been shown to inhibit Pin1 PPIase activity and suppress Pin1-mediated cell growth (Hennig et al., 1998; Uchida et al., 2003; Wildemann et al., 2006; Zhang et al., 2007; Mori et al., 2011). However, these inhibitors also suppress other PPIase activities as well as Pin1 and they have not been further studied whether they specifically affect Pin1 PTMs in the PPIase domain. Recently, two Pin1 inhibitors have been shown to directly bind to the catalytic domain, increase Pin1 degradation, and inhibit Pin1-mediated cell growth in cancer cells (Wei et al., 2015; Campaner et al., 2017; Liao et al., 2017; Zheng et al., 2017; Kozono et al., 2018). Food and Drug Administration (FDA)-approved all-trans retinoic acid (ATRA) has been identified to form salt bridges with Lys63 and Arg69 of Pin1 PPIase domain and may mimic Ser71 phosphorylation by DAPK1 because both amino acid residues are critically involved in the phosphate binding to Pin1 Ser71 (Lee et al., 2011a; Wei et al., 2015). Wei et al. (2015) showed that Pin1 levels are inversely correlated with the expression of DAPK1 in human triple negative breast cancer tissues and ATRA sensitivity is also negatively correlated with Pin1 Ser71 levels. Since DAPK1 expression is dramatically suppressed in most of solid tumors, ATRA binding to the Pin1 active site inhibits its substrate isomerase activity, thereby ultimately leading to Pin1 degradation. Another small molecule, KPT-6566 covalently binds and transfers the sulfanyl-acetate to the Cys113 residue of the Pin1 catalytic domain, and inhibits Pin1 PPIase activity (Campaner et al., 2017). Moreover, KPT-6566-B, the byproduct of KPT-6566 after Pin1 interaction, produces ROS and increases cancer cell death. Since Pin1 Cys113 oxidation abolishes Pin1 catalytic activity, KPT-6566 binding to Pin1 may have similar effects as a result of Pin1 modification.

None of drug candidates targeting Pin1 PTMs for AD has been reported yet. Instead, conformational phospho-specific antibodies targeting Thr231 of tau which is the Pin1 binding site have been developed (Nakamura et al., 2012a; Kondo et al., 2015; Albayram et al., 2017). Recently, *cis* phosphorylated Thr231 tau (p-tau), but not *trans* p-tau, has been shown to disrupt microtubule structure and axonal mitochondrial transport, spread through the brain in a prion-like fashion, and is associated with neurofibrillary tangles (Albayram et al., 2016, 2018; Lu et al., 2016). Kondo et al. (2015) generated mouse monoclonal antibodies (mAbs) specifically distinguishing *cis* from *trans* p-tau and found that *cis* mAb is able to enter neurons and effectively blocks time-dependent induction of pathological *cis* p-tau without affecting physiological *trans* p-tau, suggesting that *cis* p-tau antibody therapy may offer new approaches to treat tau-related pathologies including AD.

However, Pin1 modulator as a drug target may be challenging since both activation and inhibition of Pin1 activity may contribute to pathological conditions. Mouse develops normally in the absence of Pin1 although Pin1 KO has defects on the differentiation of neuronal stem cell, testis, and breast (Fujimori et al., 1999; Liou et al., 2002; Atchison and Means, 2003; Atchison et al., 2003; Nakamura et al., 2012b; Luo et al., 2014). Moreover, Pin1 KO mice show age-dependent neurodegeneration and Pin1 transgenic mice exhibit malignancy in breast (Liou et al., 2003; Suizu et al., 2006; Lee et al., 2009). These results indicate that targeting Pin1 or its PTMs should be carefully considered due to possible side effects depending on specific diseases and modified for designing more selective pharmaceuticals with tissue specificity and fewer off-target liabilities in the future.

## Conclusion and Perspective

Pin1 is the key protein isomerase that regulates cell growth, proliferation, cell cycle progression, apoptosis, and degeneration through the signal transduction pathways. The most important mechanism that regulates Pin1 enzymatic activity are mediated by PTMs. Pin1 consists of multiple domains with special PTM sites that are important for catalytic activation related to pathology. Pin1 PTMs affect the binding ability, stabilization and/or localization of Pin1 as well as its catalytic activity, ultimately resulting in regulating Pin1 cellular functions (Table 1). It is crucial to identify and characterize the role of PTMs in Pin1 signaling *in vivo* in future studies to determine its role as a switch between physiological and pathological conditions. Although single-site modification might precisely adjust Pin1 function only in a cell- or tissue-type-specific manner, the numerous possible combinations of different modifications could regulate Pin1 activity, enabling Pin1 to confer its various effects. Thus, the extensive cross-talk between different Pin1 modifiers, including those that mediate phosphorylation, ubiquitination, oxidation, acetylation and SUMOylation, need to be studied. Understanding the detailed degree and ratio to which modified and unmodified PTMs vary in human diseases such as cancer and AD could provide key therapeutic interventions that target Pin1. Furthermore, the discovery of novel types of PTMs in Pin1 and their potential roles are important for obtaining new insight into Pin1 related proliferation and degeneration and associated disorders. Indeed, the possibility of a Lys acetylation site in Pin1 has been identified, although the regulatory role of this acetylation of Pin1 remains to be determined (Choudhary et al., 2009; Ando et al., 2013). By elucidating the mechanism and significance of Pin1 PTMs in normal and pathological conditions, this study will provide not only a better idea of how these PTMs are induced but also a basis on which to determine whether they are viable targets for therapeutic intervention.

**TABLE 1:** Pin1 is regulated by various post-translational modifications.

Modification type	Modification site	Enzyme	Effect on Pin1	References
Phosphorylation	S16	PKA	Inhibit binding ability and nuclear localization	Lu et al., 2002
Phosphorylation	S16	Aurora A	Inhibit binding ability	Lee et al., 2013
Phosphorylation	S16	RSK2	Increase binding ability	Cho et al., 2012
Phosphorylation	S16	COT	Increase binding ability	Kim et al., 2015
Phosphorylation	S71	DAPK1	Inhibit catalytic activity and nuclear localization	Lee et al., 2011a; Kim et al., 2014; Mahoney et al., 2018
Phosphorylation	S138	MLK3	Increase catalytic activity and nuclear translocation	Rangasamy et al., 2012
Phosphorylation	S65	Plk1	Inhibit ubiquitination and increase protein stability	Eckerdt et al., 2005
SUMOylation	K6, K63	SUMO1	Inhibit binding ability and catalytic activity	Chen et al., 2013
Oxidation	C113		Inhibit catalytic activity and nuclear localization	Chen et al., 2015
Oxidation	M130, M146		Unknown	Ando et al., 2013
Acetylation	K46		Unknown	Choudhary et al., 2009; Ando et al., 2013

*PKA, cAMP-protein kinase A; RSK2, ribosomal protein S6 kinase 2; DAPK1, death-associated protein kinase 1; MLK3, mixed-lineage kinase 3; Plk1, polo-like kinase 1; SUMO1, small ubiquitin-like modifier 1.*

## Author Contributions

DC and TL conceptualized the manuscript. DC prepared the figures. LW professionally edited the manuscript. DC, LW, and TL wrote the manuscript.

## Conflict of Interest

The authors declare that the research was conducted in the absence of any commercial or financial relationships that could be construed as a potential conflict of interest.
